# The Covariate's Dilemma

**DOI:** 10.1371/journal.pgen.1003096

**Published:** 2012-11-08

**Authors:** Joel Mefford, John S. Witte

**Affiliations:** 1 Department of Epidemiology and Biostatistics, University of California San Francisco, San Francisco, California, United States of America; 2 Institute for Human Genetics, University of California San Francisco, San Francisco, California, United States of America; The University of Queensland, Australia

An important step in analyzing genetic association study data is deciding whether to adjust for covariates—those variables ancillary to the variants of interest. In particular, when testing for novel associations, should the statistical model also include known genetic or nongenetic covariates that are predictors of the trait (e.g., body mass index when studying type 2 diabetes)? Yes, if the covariates are also correlated with the primary variants but do not mediate their effects, because they may confound the genetic associations. Including them helps control bias and prevent false discoveries (Figure 1a). But the answer is less clear-cut if the covariates are not confounders.

**Figure 1:**
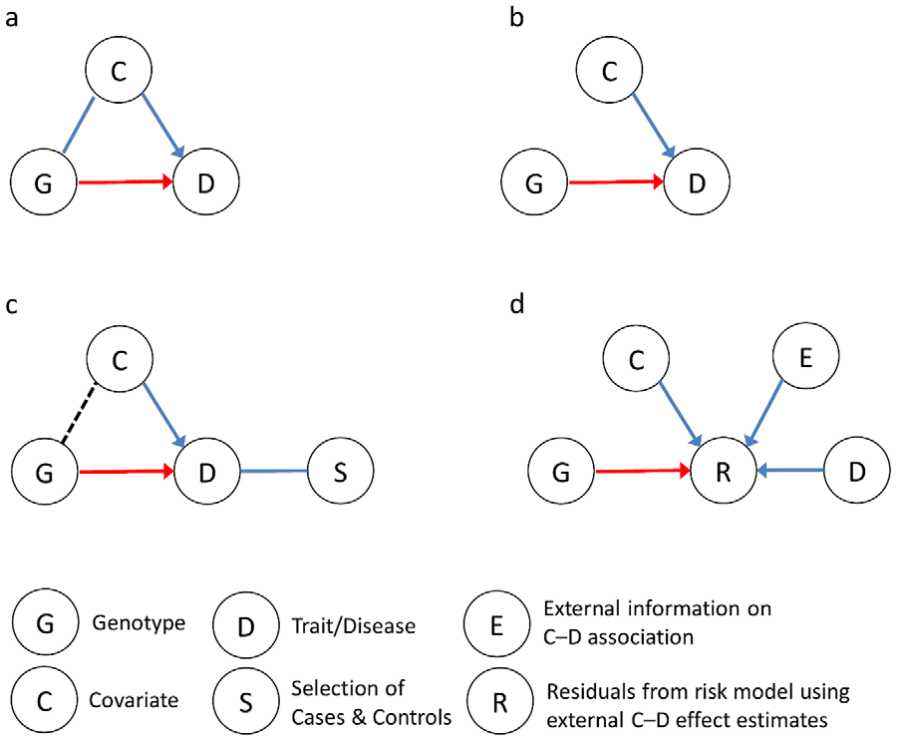
Impact of—and approaches to—including covariates in the analysis of gene–trait associations. (a) The covariate C is a confounder associated with both the trait D and the gene G but is not an intermediate on the causal path of interest between G and D. The G–D association should be assessed while controlling C. Omitting C from the analysis of the G–D association can lead to misattribution of a C–D effect to G and false discovery or biased estimates of a G–D effect. (b) The covariate C is independently associated with the trait D but not with gene G (so C is not a confounder). If the trait is quantitative or the study subjects are randomly ascertained, including C in a linear or logistic regression model will increase power to detect the G–D association. (c) If the trait is binary and the subjects are ascertained based on case-control status, the probability of selection (S) depends on G and C and induces a correlation between them. Then including C in a logistic regression model can inflate the G–D association's standard error, reducing power. Omitting C provides the most potential gain in power when C has a strong effect on D, and when D is less common [1]. (d) In Zaitlen et al.'s new approach [6] for evaluating G–D associations with case-control data, a risk model for D is developed from external information about the C–D association and observed C and D levels. Residuals from this model, R, distinguish high- and low-risk cases and controls. Then testing for G–R associations assesses genetic effects unexplained by C in a potentially more powerful manner than conventional logistic regression.

When the trait of interest is quantitative, including a nonconfounding covariate associated with the trait is often beneficial because it can explain some of the variability in the outcome, thus reducing noise and increasing power to detect novel genetic associations. On the other hand, when the trait is binary, including the covariate can actually reduce power for case-control association studies; this is shown in a recent paper by Piranen et al. [1] and previous work [2]–[5]. Fortunately, all is not lost. In this issue of *PLOS Genetics*, Zaitlen et al. [6] present a new approach that addresses this problem by leveraging information on covariates to increase power in association studies of binary traits.

## Ignorance Is Bliss…

How can ignoring covariate information increase power? Assume that we are studying the potential association between a genetic variant and a binary trait. Moreover, assume we have measured a genetic or environmental covariate associated with the trait but independent of the variant of interest in the source population, so it is not a confounder (Figure 1b). If we ascertain a random sample of study subjects, then the variant of interest and covariate will remain independent. Here, the most powerful model for assessing association *includes* the covariate (e.g., in a logistic regression model) [1]. While adding the covariate may increase the standard error of the variant association, omitting it can bias the association towards the null hypothesis of no effect and ultimately reduce power [1]–[5], [7].

However, most association studies do not select a random sample of study subjects, but rather ascertain cases and controls from the source population. This ascertainment process can create a correlation between the genetic variant and covariate in the sample, because cases will be enriched for both risk genotypes and high-risk covariate levels. Since these are independent in the source population, they will remain conditionally independent among cases or controls; but the variant and covariate will be correlated in the overall case-control sample (dashed line in Figure 1c). In the presence of this induced correlation, *omitting* the covariate from a logistic regression model may be the most powerful approach. Indeed, including the covariate could substantially increase the standard error of the genetic variant association (i.e., due to the induced correlation), resulting in a larger power loss than might arise from omitting the covariate and biasing the association towards the null hypothesis.

Pirinen et al. [1] investigate this phenomenon in detail and show that the increase in power from omitting covariates is a function of disease prevalence and effect sizes. In particular, omitting a covariate can often improve power to detect genetic effects for diseases with prevalence below 2% or as high as 10% when the covariate is a particularly strong risk factor.

## Knowledge Is Power!

Improving analyses by ignoring covariates seems counterintuitive, as they should provide some information. To extract value from covariates, Zaitlen et al. [6] developed a new method that uses existing evidence of covariate associations with the trait of interest, and trait prevalence, to increase power. This approach first builds a liability model using estimates of a covariate's independent effect in the form of trait prevalences at various levels of the covariate (e.g., type 2 diabetes prevalences by age). Then it evaluates the association between the genetic variant of interest and the liability model residuals (Figure 1d). In effect, the external information about covariate effects is used to distinguish high- and low-risk cases and controls. Tests of genetic variant associations with these quantitative residuals have more power than tests of genetic associations with the original binary trait.

The value of Zaitlen et al.'s approach is demonstrated in several data sets with case-control and case-control-covariate ascertainment, where the selection probability for an individual to join the study depends on covariate levels, such as in matched studies or those with overrepresentation of low-risk cases. While covariate-based ascertainment of cases and controls can induce selection bias that must be addressed by including the covariate in a conventional regression model [8], the new method provides a potentially powerful alternative.

The authors show by application and simulation that the liability model approach increases association test statistics by 18% and 16% in comparison with logistic regression with or without covariates, respectively. Of course, this improvement hinges on having accurate external covariate information; one could envision scenarios where the external covariate data is so poor that using this approach would actually decrease power. One could also use covariate information discerned from a given dataset, but external information may be even better. A framework to propagate uncertainties through the multistage analysis of Zaitlen et al. would be useful to assess sensitivity to the quality of published or assumed trait prevalences and covariate effects, and to the estimation errors in the formation of the liability model and in the calculation of residuals. A starting point might be to repeat the analyses for a range of covariate-specific trait prevalences that bracket the actual published or assumed values.

Zaitlen and colleagues have also developed a version of the liability model approach for when the covariates are genetic markers with known trait associations [9]. Future work might compare these novel liability methods to alternative approaches for inclusion of external information, such as Bayesian models with informative priors for the covariate effects. Moreover, schemes for weighted analyses [10] suggest other ways to potentially increase association study power.

In summary, if one undertakes a case-control association study and has information on covariates that are independent risk factors for a trait—and are not confounders—simply including them in a logistic regression model is not always the optimal approach for discovering genetic variants. Instead, more power may be gained by excluding them, by using the liability model approach of Zaitlen et al. [6], [9], or by applying other novel techniques to leverage information from such covariates.
